# bulkAnalyseR: an accessible, interactive pipeline for analysing and sharing bulk multi-modal sequencing data

**DOI:** 10.1093/bib/bbac591

**Published:** 2022-12-30

**Authors:** Ilias Moutsopoulos, Eleanor C Williams, Irina I Mohorianu

**Affiliations:** Wellcome—MRC Cambridge Stem Cell Institute, Jeffrey Cheah Biomedical Centre, University of Cambridge, CB2 0AW, UK; Wellcome—MRC Cambridge Stem Cell Institute, Jeffrey Cheah Biomedical Centre, University of Cambridge, CB2 0AW, UK; Wellcome—MRC Cambridge Stem Cell Institute, Jeffrey Cheah Biomedical Centre, University of Cambridge, CB2 0AW, UK

**Keywords:** bulk sequencing, mRNAseq, ChIPseq, epigenetics sequencing, quality checking, differential expression, gene regulatory networks, multi-omics integration

## Abstract

Bulk sequencing experiments (single- and multi-omics) are essential for exploring wide-ranging biological questions. To facilitate interactive, exploratory tasks, coupled with the sharing of easily accessible information, we present bulkAnalyseR, a package integrating state-of-the-art approaches using an expression matrix as the starting point (pre-processing functions are available as part of the package). Static summary images are replaced with interactive panels illustrating quality-checking, differential expression analysis (with noise detection) and biological interpretation (enrichment analyses, identification of expression patterns, followed by inference and comparison of regulatory interactions). bulkAnalyseR can handle different modalities, facilitating robust integration and comparison of *cis*-, *trans*- and customised regulatory networks.

## Introduction

Bulk sequencing plays a key role in modern biomedical research, as a primary resource for generating hypotheses [1]. The wide diversity of available methods, each with specific trade-offs on speed, robustness and reproducibility, hinders swift exploration of data sets. Options include bespoke analyses (through dedicated bioinformatics support), commercially available solutions (often expensive, and with limited flexibility for customisation) or fragmented pipeline-components relying on intermediary conversions of inputs/outputs [2–4].

Various pipelines/tools were developed to facilitate the analysis of bulk data; early versions focused exclusively on RNAseq data sets. A summary of some of these tools, with features contrasted to bulkAnalyseR, is presented in Supplementary Table S1. Most early tools focused on ease-of-use, and exhibited limited functionality. *Degust* [5] and *DEApp* [6] are centred on inference of differential expression (DE), only permitting few focused visualisations. *DEBrowser* [7],* iDEP* [8] and *GENAVi* [9] provide a standard analysis comprising normalisation of expression levels, DE and gene set enrichment analysis (GSEA). To the best of our knowledge, none of the existing tools go beyond this minimum standard and focus exclusively on the initial exploration of data rather than a more comprehensive analysis, including some functional interpretation of the outputs. More recent pipelines, with a wider scope are rarely self-sufficient, i.e. do no cover end-to-end analyses, and have limited appeal to researchers with restricted bioinformatics resources. *VIPER* [10] provides an end-to-end workflow starting from fastq files, but requires computing skills for setting up analyses and offers restricted scope for output customisation with user-controlled parameters (thresholds). *BioJupies* [11] is simpler to run and boasts a larger selection of outputs; however, it restricts the customisation of individual components, e.g. to selecting genes; additionally, fewer options for the biological interpretation of outputs are available. Critically, neither offers support for complex DE tasks, beyond simple pairwise comparisons, limiting the biological interpretations of complex experimental designs. *Searchlight* [12] alleviates some of these issues, focusing on an interactive exploration of results; the inputs are tables of DE genes, relying on several a priori steps [quality checks (QCs) and DE inference]. It also assumes expertise for setting up the environment and retrieving outputs; moreover, the resulting plots require additional tweaking to meet publication requirements.

Current state-of-the-art analyses go beyond generating lists of DE genes, tapping into causal (gene interaction) inference, employing single- or multi-omics inputs [13, 14]; however, most gene regulatory network (GRN) inference tools are standalone. An early effort linking prediction and visualisation [15] underlines the lack of and need for direct, customisable bridges between analyses and biological interpretations. Visualisation tools such as *Cytoscape* [16] facilitate hypothesis generation, but require external definitions of the networks. Moreover, GRN inferences are often performed on the entire data set, generating complex, difficult-to-interpret networks [17] and lacking in robustness because of intrinsic variability [18]. *GeNeCK* [19] attempts, in a web-based setting, to link GRN inference and interpretation, but is decoupled from other steps such as DE analysis.

bulkAnalyseR enables the analysis of single- and multi-omics bulk-sequencing data, in an interactive Shiny interface, with several customisable components, from noise detection [18] to the identification of patterns [20] and GRN prediction [4]; the latter accommodates *cis*-, *trans*- and custom interactions. The setup facilitates a seamless transition to publication-ready figures without additional bioinformatics support. The resulting web-app is easily publishable, incentivising open and reproducible research.

## Materials and methods

### Pipeline overview

**Figure 1:**
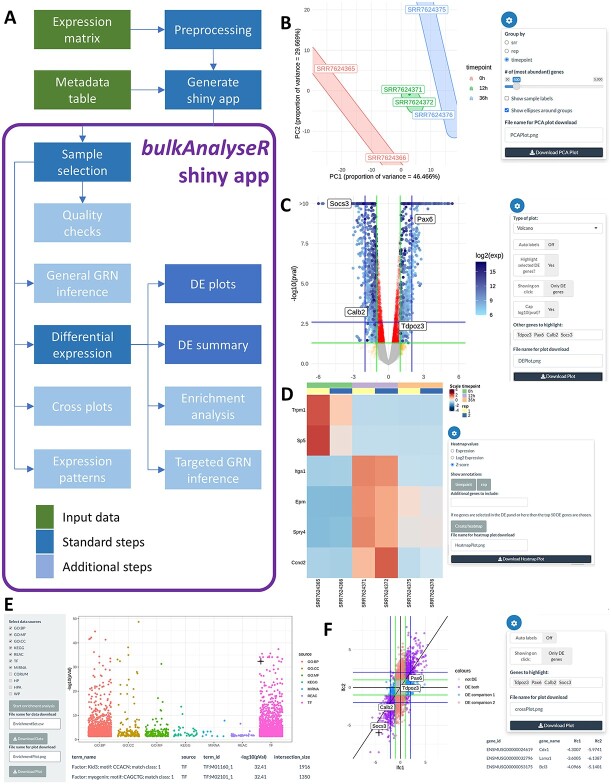
Workflow and examples of bulkAnalyseR summary plots on bulk mRNAseq data [13]. (**A**) Workflow diagram of the main components of the pipeline and package; further details are presented in Supplementary Figure S1. (**B**) PCA plot of all samples illustrating sample similarity. (**C**) Volcano plot showcasing DE genes between the 0 and 12h samples. (**D**) Heatmap of *Z*-scores across samples for the top differentially expressed genes. (**E**) Enrichment analysis results for the DE genes (enriched terms ordered are by significance). (**F**) Cross-plot comparing two DE outputs (log_2_(FC) from 0 versus 12h on the *x*-axis and 0 versus 36h on the *y*-axis).

From a user perspective, bulkAnalyseR relies on two central functions: *preprocessExpressionMatrix* and *generateShinyApp*. The former handles all standard pre-processing, i.e. the denoising of the input expression matrix using noisyR [18]; the normalisation of expression levels (using e.g. quantile normalisation [21], DESeq2 [3] or TMM [2]) is also included. Using the pre-processed object, the *generateShinyApp* function creates a shiny app, linking the expression matrix and user-defined metadata table. For mRNAseq inputs, the resulting app comprises 10 panels (Figure 1), covering input visualisation and quality checking, DE inference followed by visualisation and comparison of results, GSEA, flexible inference of GRNs and their dynamic across conditions and the identification of expression patterns. Further details on individual panels are presented in the Results and Discussion section.

The pipeline is also agnostic to other modalities, e.g. ChIPseq peak amplitudes or ncRNAs (miRNA) expression, both illustrated as case studies. For non-RNAseq modalities, all panels apart from GSEA, which cannot be defined on non-protein-coding genes, are available. bulkAnalyseR also enables the integration of multiple modalities, through combined GRNs. Options focus on the expected type of interaction: (i) *cis*-regulatory interactions for which users supply a set of coordinates for each modality (e.g. protein-coding genes and peak locations for mRNAseq/ChIPseq integration) and (ii) *trans*-regulatory interactions where users provide only expression matrices with common columns. Moreover, the custom integration option enables users to annotate GRNs with any available external information, e.g. annotating genes with known miRNA targets using miRTarBase [22].

### Data pre-processing

#### RNAseq modality

The single-omics mRNAseq functionality was exemplified using several mRNAseq samples from the Yang *et al*. [13] study (namely, 0, 1, 6, 12, 24, 36, 48 and 72h post-stem cell induction). The raw fastq files were downloaded from GEO (accession GSE117896). Initial QCs were performed using fastQC (version 0.11.8) and summarized with multiQC (version 1.9) [23]. Alignment to the *Mus musculus* genome was performed using STAR (version 2.7.0a) with default parameters [24]; the count matrices were generated using featureCounts (version 2.0.0) [25] against exon annotations from Ensembl (assembly GRCm38.p6).

#### Epigenetic (ChIP) modality

The ChIPseq modality (for showcasing *cis*-interactions) was illustrated using several samples from the Yang *et al*. study; identical time points as for the mRNAseq samples were selected. Alignment to the *Mus musculus* genome was performed with bowtie2 v2.4.2 (single-end mode, local mode default parameters) [26]; peak calling was performed using macs2 2.2.7.1 [27]. Peak aggregation across samples (for generating a unified expression matrix) was performed on the union of matched peaks. Peaks are matched if, per peak, its midpoint in one sample is within the peak genomic range in the other sample. Using pyBigWig 0.3.17 (deeptools, version 3.5.0 [28]), the corresponding amplitudes for these peaks were calculated (and scaled on the read length). The bigwig files were produced using bamCoverage (deeptools) with default parameters.

#### Non-coding RNA modality

To showcase *trans*-regulatory interactions, we focused on a mRNA/microRNA case study [29]; the data set describes intervertebral disc degeneration and comprises three control and three treatment (IDD) samples. The raw fastq files were downloaded from GEO (accession GSE167199). The mRNA samples were aligned to the *Homo sapiens* reference genome using STAR (version 2.7.0a) with default parameters [24]; the count matrices were generated using featureCounts (version 2.0.0) [25] against exon annotations from Ensembl (assembly GRCh38.p13). Small RNA (microRNA) samples were aligned against *Homo sapiens* mature microRNAs from miRBase [30] using PaTMaN [31] with zero edits and zero gaps. The mature microRNA expression was calculated as the number of exact copies of the miRNA sequence.

#### Spatial transcriptomics using 10x Visium

To illustrate the flexibility of bulkAnalyseR, we present a case study on spatial expression data. We focus on the stxBrain anterior1 data set (https://support.10xgenomics.com/spatial-gene-expression/datasets/1.0.0/V1_Mouse_Brain_Sagittal_Anterior) pre-processed using Seurat [32]. From the cortex region and other layers annotated in the Seurat spatial vignette we selected the Top 5 spots (ranked by score predicted by the authors of the vignette). We focused on the L2/3 IT, L4, L5 IT, L6 IT regions. The transcriptomic signatures (as counts) for the 20 spots were denoised using noisyR [18] and quantile normalised [21]. Spots corresponding to each layer were considered replicates.

#### Customised interactions

To illustrate customised interactions, we integrated the outputs from the enrichment step, and included the option to decorate a GRN with manually curated entries from miRTarBase [22], for posttranscriptional regulation, and TRANSFAC [33], for transcriptional regulation.

#### DE and biological interpretation steps

For the case studies presented in the manuscript, the noise correction was performed using the counts approach from the noisyR package [18]. The normalisation was performed using (a) the preprocessCore package for quantile normalisation, (b) by scaling each value by 1 million divided by the reads in each sample for RPM, (c) the DESeq2 package for its internal normalisation, (d) the edgeR package for TMM and (e) by dividing each value by the median expression in each sample for the median normalisation.

The DE call is performed using the DESeq2 [3] or edgeR [2] pipelines, using default parameters (with data pre-normalised), on a comparison of the two conditions specified. The enrichment analysis is based on the gprofiler2 package [34] with a custom background of all genes expressed in the data set. The GRN inference is performed using the GENIE3 package [4] focusing on user-defined regulatory targets. When more than one network is generated, edges appearing in more than one network are highlighted. An UpSet plot [35] summarises the intersections and specific differences of the GRNs.

## Results and discussion

**bulkAnalyseR** provides an accessible, yet flexible end-to-end framework for analysing bulk-sequencing data sets, without relying on prior programming expertise (Figure 1 and Supplementary Figure S1). It generates a shareable Shiny app in two lines of code; all resulting plots and tables can be downloaded individually and the underlying code for generating the outputs can be easily reproduced.

### Inputs and pre-processing

The inputs for bulkAnalyseR are an expression matrix and a metadata table. The former contains counts per genes across samples (e.g. generated from BAM alignments with featureCounts [25]). The metadata table contains additional experimental information and represents the starting point for the DE analyses/comparisons. The pre-processing step (*preprocessExpressionMatrix* function) handles the counts-based noise detection using noisyR [18], which outputs a denoised, un-normalised expression matrix (Supplementary Figure S2). Several options are available for normalisation [36]: quantile (default [21]), per-total [37], TMM [2] and DESeq2 [3]. Integration with, or replacement by, other pre-processing steps is also possible, as any expression matrix can be passed onto the next step. Next, *generateShinyApp* checks the compatibility of inputs, and the expression matrix (denoised, normalised, by default), and a Shiny app is created. The resulting app is standalone and shareable, e.g. via online platforms like shinyapps.io (https://docs.rstudio.com/shinyapps.io/) (further details are provided in Supplementary Figure S7). This setup increases access and promotes reproducibility of bioinformatics analyses.

### Interactive visualisation of DE

A single-omics instance comprises several tabs: (a) QCs include a Jaccard Similarity Index heatmap and a PCA dimensionality reduction, with groups based on the metadata information (Figure 1B and Supplementary Figure S4A and B). This enables a high-level overview of the similarity across samples, reflecting the experimental design. (b) The DE tab includes DE summaries across selected comparisons, performed using edgeR [2] and DESeq2 [3] with customisable parameters (significance and fold-change thresholds are user-adjustable); the outputs are tables of DE genes (Supplementary Figure S4C). (c) The DE results can be visualised in the Volcano/MA and DE summary panels that promote an interactive exploration of the data (Figure 1C and Supplementary Figure S4E, F and H). (d) The interpretation of the DE genes commences with the enrichment tab, which overviews a GSEA using g:Profiler [34]. The GSEA is performed on GO terms, KEGG and Reactome pathway terms, and regulatory features (miRNAs [22] and transcription factors [33]) Figure 1E. Further information is obtained by (e) identifying simplified patterns that may underline regulatory interactions (Supplementary Figure S3I) and (f) comparing DE results in cross-plots (Figure 1F and Supplementary Figure S4D).

The differences observed between frequently used DE inference pipelines (either because of normalisation details or criteria for the DE call), pertinently discussed in Li *et al*. [38] and Moutsopoulos *et al*. [18], are assessed in Supplementary Figure S3 on the Yang *et al*. data set. The variability in results is significantly reduced by addressing the technical noise [18]. For this case study, we highlight these differences by contrasting the DESeq2 and edgeR DE outputs, when the pipelines are run on the same inputs (noisy and denoised matrices are assessed, alongside different normalisation methods).

### Visualisation of gene regulatory networks

**Figure 2:**
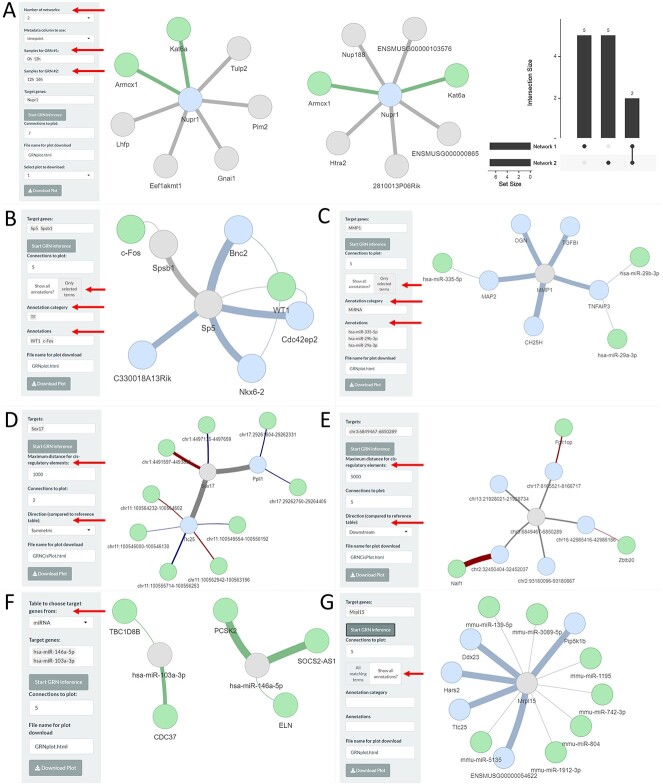
Examples of GRN analyses. (**A**) Yang *et al*. case study, visualisation of Top 7 connections of gene *Nupr1*; the inference was performed on 0 versus 12h samples (left) and 12 versus 36h samples (middle). The width of the edges is proportional to the weight from the adjacency matrix. Target genes are shown in blue; common genes between the two networks are highlighted in green. The overlapping genes between the two networks are summarised in an UpSet plot (right). (**B**) GRN inferred on 0 versus 12h DE genes augmented with enriched TF information (green, from gprofiler2). (**C**) Li *et al*. [29] case study, GRN network inferred on control versus IDD DE genes for the mRNAseq modality, augmented with enriched microRNAs (green, from gprofiler2). (**D**) Yang *et al*. case study, GRN network inferred for *Sox17* for 0h, 12h and 36 h for the mRNAseq modality with *cis*-integration of the corresponding ChIpseq modality; in green the H3K4me3 ChIP peaks within 1 kb of target genes are shown. Upstream peaks are highlighted with blue edges; downstream peaks are highlighted with red edges. (**E**) GRN network inferred on the H3K4me3 modality, with *cis*-integration of genes (green) within 1 kb downstream from the selected peaks. (**F**) Li *et al*. [29] case study, *trans*-integrated GRN, for mRNA and miRNA modalities; the GRN is miRNA-centric. (**G**) Yang *et al*. case study, GRN inferred on *Mrpl15* for 0, 12 and 36h mRNAseq modality with customised miRTarBase integration (miRNAs linked to the selected mRNA are highlighted in green).

Data exploration continues by inferring networks of regulatory interactions (GRN tab), using GENIE3 [4]. To allow the visualisation of changes in topology or strength of the interaction (in terms of co-expression) across different subsets of samples, up to four inferences can be performed simultaneously. Focus genes can be selected from the set of non-constant genes; to increase the robustness of this analysis, we recommend using up to three indirect interactions from the focus genes (Figure 2A and Supplementary Figure S4J). An UpSet plot summarises the common vertices across the resulting networks.

### Case studies of data sets with additional characteristics

#### Temporal and Spatial data sets

bulkAnalyseR is versatile (and agnostic) in terms of the input of data. The pipeline can cover a range of conditions, from various treatment groups, tissue types, time points and spatial information. To underline this flexibility, we showcase the full time series from the Yang *et al*. [13] study. In Supplementary Figure S7A–F, we contrast the interpretation of outputs obtained on a variable number of time points and illustrate the ability of the bulkAnalyseR pipeline to summarise patterns. In this example, the smooth progression throughout the time course (QC panel, subplot A) mirrors the information in the patterns panel (subplot D). Moreover, using the higher number of time points, we uncover patterns (subplot D) not captured using fewer time points (subplots E, F). Using bulkAnalyseR, various combinations of samples can be compared and contrasted, on the full sets of outputs, without recreating the app. To further emphasise its versatility, we applied the bulkAnalyseR pipeline to a few spots from five different cortical layers from a 10x Visium data set (Supplementary Figure S7G–I). Analogously as for the previous case study, we capture the transcriptomic changes and patterns through space and enable users to query genes of interest and visualise expression through layers and regions. The example apps for both case studies are publicly available (temporal and spatial data sets).

#### Processing of other single modalities

The input, based on an expression matrix, confers flexibility to bulkAnalyseR. Other sequencing data sets, which can be summarised in an expression matrix, can be processed using this pipeline (we illustrate a case study of H3K4me3 ChIPseq data, Supplementary Figure S5, and of a small RNA data set, with a focus on mature microRNA expression levels, Supplementary Figure S6). For these analyses, all steps except the GSEA, which cannot be defined on non-protein-coding genes, are available. The versatility of integrating multiple modalities or different experiments (of the same or mixed modalities) in the same framework is a promising first, interactive step to guiding mechanistic studies (e.g. multi-omics GRN inference).

#### Multi-omics GRN integration

bulkAnalyseR also permits the integration of multiple modalities for inferring regulatory interactions (i.e. *cis*-, *trans*- and user-defined). Two inputs are required for the ‘*cis*-interactions’, which undergo soft/late integration: the focal and non-focal modalities comprising elements located in close genomics proximity (e.g. 1 kb, user-defined parameter) are used to augment the focal GRN. For example, the RNA-centric GRN can be augmented with information on *cis*-elements such as ChIPseq peaks (Figure 2D); the two modalities are interchangeable (Figure 2E). The proximity parameter can encompass entries from the non-focal data set which are upstream, downstream or both, of the selected entries in the focal data. For the ‘*trans*-interactions’, the two inputs undergo complete/early integration, i.e. the two expression matrices, with identical columns/conditions, are concatenated. The GRN is inferred on the combined expression matrix; nodes are coloured according to the original source of the element (Figure 2F). Given that the GRN inference is agnostic of expression amplitude, focusing just on variational patterns, no additional batch correction or over-normalisation is performed. The selected targets for the localised GRN can be selected from either input. Custom, user-defined interactions can be added/visualised; these rely on external information related to the vertices in the inferred GRN. Pre-loaded examples include adding miRNAs targeting protein-coding genes, retrieved from miRTarBase [22] (Figure 2B) and interactions with transcription factors (Figure 2C), retrieved from gprofiler2 [34]. Users can also embed their own customised interactions, supplied as a table (Figure 2G). Example apps illustrating multi-omics GRN integration are Yang *et al*. mRNA/ChIP integration and Li *et al*. mRNA/microRNA integration. A vignette detailing the integration of multi-omics and external data can be found here.

## Conclusions

The aim of bulkAnalyseR is to enhance the interactive interrogation of single- and multi-omics data; additionally, the ability to share a stable instance of an analysis with the community has the potential to ease communication between research groups and generate new hypotheses that extend beyond the initial purpose of data sets. Moreover, the seamless integration of all steps in an end-to-end approach, from early quality control checks through to publication-ready figures, assists with data mining throughout the life cycle of an analysis.

More importantly, bulkAnalyseR provides the flexibility to integrate several modalities and data sets, and incorporate external databases through standard enrichment analysis, multi-omics integration and more customisable pipelines. This generates a valuable/reliable starting point towards more complex questions that may pave the road towards a better mechanistic understanding of the cause(s) behind the observed differences in expression.

**Key Points**

- bulkAnalyseR integrates state-of-the-art approaches for noise correction, inference of differential expression and interpretation of results, using an expression matrix as the starting point (pre-processing functions are available as part of the package).
- bulkAnalyseR can handle different modalities, facilitating robust integration and comparison of *cis*-, *trans*- and customised regulatory networks. This feature allows for the first time the interactive assessment of network dynamics both across single modalities, and in the context of regulatory features.
- bulkAnalyseR generates interactive and shareable Shiny apps, in two lines of code, promoting collaborations between groups and facilitating further exploration of data sets, beyond the life-span of original projects.

## Supplementary Material

bulkAnalyseR_supp_bbac591

## Data Availability

bulkAnalyseR is available on CRAN and GitHub, with extensive documentation and usage examples (https://github.com/Core-Bioinformatics/bulkAnalyseR, https://cran.r-project.org/web/packages/bulkAnalyseR/).

## Author Biographies

**Ilias Moutsopoulos** (MSc in system biology, University of Cambridge, UK) is a PhD student in the Core Bioinformatics group; his interests revolve around a data-driven inference of gene regulatory networks dynamics.

**Eleanor C. Williams** (MSc in maths and computer science, University of Oxford, UK) is a research assistant/PhD student in the Core Bioinformatics group; her interests are focused on regulatory interactions driven by non-coding RNAs and integration of multi-modal data sets.

**Irina I. Mohorianu** (PhD in computer science, Norwich Research Park, UK) leads the Core Bioinformatics group, as head of Bioinformatics/Scientific Computing; her interests span applications of machine learning/data science approaches for high throughput biological data sets.
